# Analysis of the Nutritional Value of the Diets Presented in Women’s and Sports Magazines before and during the COVID-19 Pandemic

**DOI:** 10.3390/ijerph19169859

**Published:** 2022-08-10

**Authors:** Dominika Głąbska, Maria Janowska, Ewa Bartosz, Dominika Guzek

**Affiliations:** 1 Department of Dietetics, Institute of Human Nutrition Sciences, Warsaw University of Life Sciences (WULS-SGGW), 159C Nowoursynowska Street, 02-776 Warsaw, Poland; 2 Department of Food Market and Consumer Research, Institute of Human Nutrition Sciences, Warsaw University of Life Sciences (WULS-SGGW), 159C Nowoursynowska Street, 02-776 Warsaw, Poland

**Keywords:** menu, diet, magazine, women’s magazine, sports magazine, nutritional value, low-calorie diet, COVID-19 pandemic

## Abstract

For consumers, among the most important sources of information related to nutrition are popular journals and magazines, including women’s and sports, but the diets presented there may lead to unhealthy weight-control behaviors. The aim of the study was to assess the nutritional value of regular and low-calorie diets presented in Polish women’s and sports magazines before and during COVID-19 pandemic. The study was based on two popular Polish magazines—one women’s magazine and one sports magazine, which regularly present various types of diets. The nutritional value of all the diets published from January 2014 to May 2022 was analyzed. The total number of included single-day menus was *n* = 119, while for analysis they were stratified based on: type of magazine (published in the women’s magazine *n* = 41 and in the sports magazine *n* = 78), year of publication (before the COVID-19 pandemic *n* = 78 and during *n* = 41), and type of diet (regular *n* = 61 and low-calorie *n* = 58). The analysis included the energy value and nutritional value. For the type of magazine, the diets published in the sports magazine were characterized by a higher intake of fat (*p* < 0.0001 for intake in grams and in % of energy) and calcium (*p* = 0.0330), whereas the diets published in the women’s magazine were characterized by a higher intake of carbohydrates (*p* = 0.0226 for intake in grams, and *p* = 0.0002 for intake in % of energy) and fiber (*p* = 0.0163). For the year of publication, the diets published during the COVID-19 pandemic were characterized by a higher intake of protein (*p* = 0.0166 for intake in grams), sodium (*p* = 0.0465), calcium (*p* < 0.0001), vitamin D (*p* = 0.0197), vitamin B6 (*p* = 0.0207), and vitamin B12 (*p* = 0.0277), whereas the diets published before the COVID-19 pandemic were characterized by a higher intake of carbohydrates (*p* = 0.0243 for intake in % of energy). For the type of diet, the regular diets were characterized by a higher energy value (*p* = 0.0020), as well as by a higher intake of fat (*p* = 0.0162 for intake in grams), carbohydrates (*p* = 0.0390 for intake in grams), mono- and oligosaccharides (*p* = 0.0084 for intake in % of energy), fiber (*p* < 0.0001), magnesium (*p* = 0.0323), iron (*p* = 0.0307), and vitamin B6 (*p* = 0.0204). The nutritional value of the diets presented in the Polish women’s and sports magazines was not justified by the type of magazine or type of diet, associated with the target group, which may cause the following of improperly balanced diets. However, the changes in the typical nutritional value of diets presented in the Polish women’s and sports magazines during the COVID-19 pandemic were justified by some specific needs for the prevention and treatment of COVID-19.

## 1. Introduction

According to the World Health Organization (WHO), no European country is on track to stop the rising rates of obesity by 2025 [1]. Moreover, the global prevalence of people who are overweight and obese has nearly tripled since 1975 [2]. At the same time, the COVID-19 pandemic has worsened the observed situation associated with excessive body mass [3] not only for adults but also for adolescents [4].

Especially for female individuals, unfavorable obesogenic nutritional behaviors were observed during the COVID-19 pandemic, resulting from a higher frequency of emotional eating [5] and lower self-regulation of eating behaviors compared to males [6]. On the other hand, women generally present as more conscious of nutrition than men, as they tend to purposively avoid foods perceived as unhealthy [7]. Similarly, they are more likely than men to relate their self-esteem to their body mass, which results in their common efforts to reduce body mass [8].

A low-calorie diet, combined with physical activity, to obtain body mass reduction in individuals who are overweight or obese is crucial to observe beneficial health effects [9]. This results from the fact that increased body mass is associated with the accumulation of abnormal or excessive fat or adipose tissue in body, linked to metabolic abnormalities and the consequent risk of cardiovascular diseases [10], type 2 diabetes [11], as well as cancer-associated mortality [12]. Taking this into account, a global strategy to prevent and treat overweight and obesity is necessary [13], while governments, international organizations, nonprofit organizations, and the private sector need to contribute complementary actions [14].

However, for consumers, among the most important sources of information related to health and nutrition are popular journals and magazines, including women’s and sports magazines [15]. Magazines are among the most preferred media for health information, including nutrition and diets, mostly low-calorie diets [16]. 

Nonetheless, for females, frequent reading of magazine articles about weight loss predicts unhealthy weight-control behaviors, which is not the case for males [17]. This may be associated with the negative influence of magazines on body image as well as on the frequency of disordered eating behaviors [18]. At the same time, the content of magazines frequently is not consistent, as it alternately promotes excessive consumption, accompanied by cooking, and restrained consumption, accompanied by dieting [19]. Last but not least, the communication to reduce consumption and to lose weight is not addressed specifically to individuals with excessive body mass, and it may cause females with a normal body mass to follow low-calorie diets as well [20].

Considering the described situation, the aim of the study was to assess the nutritional value of regular and low-calorie diets presented in the Polish women’s and sports magazines before and during the COVID-19 pandemic.

## 2. Materials and Methods

### 2.1. Ethical Issues and Study Design

The study included assessment of widely available diets published in Polish women’s and sports magazines; so, based on the goals of the ethics committee, the conducted study did not correspond to any area that would require approval of the ethics committee [21]. The conducted study did not use any human or animal data, while neither patients nor any other individuals were included, and no hospital, clinic, or any other information gathered within routine management was used [22].

The study was based on two popular Polish magazines, published in a printed version—one women’s magazine (‘Świat Kobiety’—ang. ‘Women’s World’) and one sports magazine (‘Women’s Health’), which regularly present various types of diets and have been published at least since 2014. The nutritional value of all the diets published from January 2014 to May 2022 was assessed and analyzed.

### 2.2. Inclusion of Diets

The magazines were chosen based on the following criteria:

− Women’s magazine or sports magazine;
− Published in Polish;
− Commonly available in the whole country;
− Published at least from 2014 to 2022;
− Presented diets/menus at least three times per year;
− Presented various diets/menus with specified dishes and specified amounts of food products.

Based on the described criteria, one women’s magazine (‘Świat Kobiety’ (ang. ‘Women’s World’)—https://bauer.pl/czasopismo/swiat-kobiety/, accessed on 1 July 2022) and one sports magazine (‘Women’s Health’—https://www.womenshealth.pl/, accessed on 1 July 2022) were chosen. The analysis of volumes was conducted based on the resources of the Polish National Library, while all volumes published in the period from January 2014 to May 2022 were reviewed to choose menus to analyze.

The diets were chosen based on the following criteria:

− Diet published in ‘Świat Kobiety’ or ‘Women’s Health’ in the period from January 2014 to May 2022;
− Diet published either as a single-day menu or multiple-day menu;
− Diet for a general population of healthy individuals, while any additional information was allowed (e.g., low-calorie diet, vegetarian diet, antioxidative diet, etc.);
− Diet published with specified dishes and specified amounts of food products, while amounts of food products were allowed to be presented either in grams or as household measures.

All the diets selected based on the presented criteria were included to further analysis. Multiple-day menus were divided into multiple single-day menus, to analyze each of them separately. If a multiple-day menu was presented as a list of multiple options for specific dishes to be chosen and combined, they were combined randomly to constitute multiple single-day menus.

The final number of included single-day menus was *n* = 119, while for further analysis, they were stratified based on the following criteria:

− Type of magazine—diets published in the women’s magazine (*n* = 41) and diets published in the sports magazine (*n* = 78);
− Year of publication—diets published before the COVID-19 pandemic (*n* = 78) and diets published during the COVID-19 pandemic (*n* = 41);
− Type of diet—diets not presented as low-calorie (*n* = 61) and diets presented as low-calorie (*n* = 58).

### 2.3. Assessment of Diets

The included single-day menus were assessed by a professional dietitian, while the energy value and nutritional value were calculated for each diet according to a commonly applied procedure.

If a single-day menu used household measures instead of presenting amounts of food products or dishes in grams, the household measures were translated into serving sizes in grams using the Polish Atlas of Food Products and Dishes Portion Size [23], presenting typical serving sizes used in Poland.

The energy value and nutritional value were calculated separately for each single-day menu (daily intake planned within the diet), using Polish professional dietician software Dieta 5.0 (National Food and Nutrition Institute, Warsaw, Poland). The used software was based on data from the Polish database of the energy value and nutritional value of food products and dishes [24].

The analysis included the energy value of the single-day menus, as well as the following elements of nutritional value:

− Macronutrients: protein (g), fat (g), carbohydrates (g), cholesterol (mg), fiber (g), protein (% of energy value of the diet), fat (% of energy value of the diet), carbohydrates (% of energy value of the diet), and mono- and oligosaccharides (% of energy value of the diet);
− Minerals: sodium (mg), potassium (mg), calcium (mg), phosphorus (mg), magnesium (mg), iron (mg), zinc (mg), copper (mg), and iodine (µg);
− Vitamins: vitamin A (µg retinol equivalents), vitamin D (µg), vitamin E (mg α-tocopherol equivalents), vitamin B1 (mg), vitamin B2 (mg), niacin (mg), vitamin B6 (mg), folate (µg), vitamin B12 (µg), and vitamin C (mg).

### 2.4. Statistical Analysis

The normality of distribution was verified using the Shapiro–Wilk test, and further comparisons were conducted based on the distributions. For parametric distributions, the Student’s *t*-test was used, while for nonparametric distributions, the Mann–Whitney *U* test was used. 

*p* ≤ 0.05 was indicated as statistically significant, and the analyses were conducted using Statistica 13.3 (StatSoft Inc., Tulsa, OK, USA).

## 3. Results

The energy value and macronutrient intake within the assessed single-day menus, stratified by type of magazine, for the diets published in the women’s magazine and the diets published in the sports magazine are presented in Table 1. The energy value of the diets did not differ between the diets published in the women’s magazine and the diets published in the sports magazine (*p* > 0.05). At the same time, differences were observed for fat (a higher intake for the diets published in the sports magazine; *p* < 0.0001 for intake in grams and in % of energy), carbohydrates (a higher intake for the diets published in the women’s magazine; *p* = 0.0226 for intake in grams, and *p* = 0.0002 for intake in % of energy), and fiber (a higher intake for the diets published in the women’s magazine; *p* = 0.0163).

The mineral intake within the assessed single-day menus, stratified by type of magazine, for the diets published in the women’s magazine and the diets published in the sports magazine is presented in Table 2. The intake of the majority of minerals did not differ between the diets published in the women’s magazine and the diets published in the sports magazine (*p* > 0.05); differences were only observed for calcium (a higher intake for the diets published in the sports magazine; *p* = 0.0330).

The vitamin intake within the assessed single-day menus, stratified by type of magazine, for the diets published in the women’s magazine and the diets published in the sports magazine is presented in Table 3. The intake of the assessed vitamins did not differ between the diets published in the women’s magazine and the diets published in the sports magazine (*p* > 0.05).

The energy value and macronutrient intake within the assessed single-day menus, stratified by year of publication, for the diets published before the COVID-19 pandemic and the diets published during the COVID-19 pandemic are presented in Table 4. It was observed that the energy value of the diets did not differ between the diets published before the COVID-19 pandemic and the diets published during the COVID-19 pandemic (*p* > 0.05). At the same time, differences were observed for protein (a higher intake for the diets published during the COVID-19 pandemic; *p* = 0.0166 for intake in grams) and carbohydrates (a higher intake for the diets published before the COVID-19 pandemic; *p* = 0.0243 for intake in % of energy).

The mineral intake within the assessed single-day menus, stratified by year of publication, for the diets published before the COVID-19 pandemic and the diets published during the COVID-19 pandemic is presented in Table 5. The intake of the majority of minerals did not differ between the diets published before the COVID-19 pandemic and the diets published during the COVID-19 pandemic (*p* > 0.05); differences were observed only for sodium (a higher intake for the diets published during the COVID-19 pandemic; *p* = 0.0465) and calcium (a higher intake for the diets published during the COVID-19 pandemic; *p* < 0.0001).

The vitamin intake within the assessed single-day menus, stratified by year of publication, for the diets published before the COVID-19 pandemic and the diets published during the COVID-19 pandemic is presented in Table 6. The intake of the majority of vitamins did not differ between the diets published before the COVID-19 pandemic and the diets published during the COVID-19 pandemic (*p* > 0.05); differences were observed only for vitamin D (a higher intake for the diets published during the COVID-19 pandemic; *p* = 0.0197), vitamin B6 (a higher intake for the diets published during the COVID-19 pandemic; *p* = 0.0207), and vitamin B12 (a higher intake for the diets published during the COVID-19 pandemic; *p* = 0.0277).

The energy value and macronutrient intake within the assessed single-day menus, stratified by type of diet, for the regular diets (not presented as low-calorie) and the diets presented as low-calorie are presented in Table 7. The energy value of the diets differed between the regular diets and the low-calorie diets, which was in agreement with the declared decreased energy value (a higher energy value for the regular diets; *p* = 0.0020). Taking this into account, a higher intake of all assessed nutrients (except for those assessed as % of energy) was expected for the regular diets. At the same time, differences were observed for fat (a higher intake for the regular diets; *p* = 0.0162 for intake in grams), carbohydrates (a higher intake for the regular diets; *p* = 0.0390 for intake in grams), and fiber (a higher intake for the regular diets; *p* < 0.0001). However, differences were also observed for mono- and oligosaccharides for intake in % of energy (a higher intake for the regular diets; *p* = 0.0084).

The mineral intake within the assessed single-day menus, stratified by type of diet, for the regular diets (not presented as low-calorie) and the diets presented as low-calorie is presented in Table 8. The intake of the majority of minerals did not differ between the regular diets and the low-calorie diets (*p* > 0.05); differences were observed only for magnesium (a higher intake for the regular diets; *p* = 0.0323) and iron (a higher intake for the regular diets; *p* = 0.0307).

The vitamin intake within the assessed single-day menus, stratified by type of diet, for the regular diets (not presented as low-calorie) and the diets presented as low-calorie is presented in Table 9. The intake of the majority of vitamins did not differ between the regular diets and the low-calorie diets (*p* > 0.05); differences were observed only for vitamin B6 (a higher intake for the regular diets; *p* = 0.0204).

## 4. Discussion

Within the conducted study, all the assessed variables (type of magazine, year of publication, and type of diet) were associated with the energy value or nutritional value of the diets. Taking into account the type of magazine, the diets published in the sports magazine were characterized by a higher intake of fat and calcium, whereas the diets published in the women’s magazine were characterized by a higher intake of carbohydrates and fiber. Taking into account the year of publication, the diets published during the COVID-19 pandemic were characterized by a higher intake of protein, sodium, calcium, vitamin D, vitamin B6, and vitamin B12, whereas the diets published before the COVID-19 pandemic were characterized by a higher intake of carbohydrates. Taking into account the type of diet, the regular diets were characterized by a higher energy value, as well as by a higher intake of fat, carbohydrates, mono- and oligosaccharides, fiber, magnesium, iron, and vitamin B6.

The nutritional recommendations for athletes practicing any sport are developed by international organizations, Ministries of Sports, and researchers, while they are dedicated to individuals practicing specific sports [25], so there are, e.g., recommendations for runners [26], bodybuilders [27], or football players [28]. However, there are also general nutritional recommendations or guidelines for individuals engaging in increased physical activity [29], as it is well known that physical activity, independent of the discipline, results in specific metabolic changes [30] and may be associated with increased nutritional requirements [31]. 

Considering this fact, it may have been expected to observe a higher intake of specific nutrients for the diets published in the sports magazine. However, while assessing the differences between the diets published in the sports magazine and the diets published in the women’s magazine, the differences were not justified as expected. The diets published in the sports magazine, compared with those published in the women’s magazine, were characterized by a higher intake of fat but a lower intake of carbohydrates. Taking this into account, the macronutrient proportions of diets in the sports magazine were altered, including an increased share of fat (39% of energy value, compared with 28% in the women’s magazine) and a decreased share of carbohydrates (42% of energy value, compared with 50% in the women’s magazine). Such proportions are not justified for individuals increasing their physical activity, as they are not in agreement with the general dietary recommendations for healthy individuals by the Food and Agriculture Organization of the United Nations (FAO) [32] and WHO [33], indicating a reduction in fat intake to 30%. Moreover, for athletes, it is indicated that despite some potential benefits, low-carbohydrate and high-fat diets may contribute to increased central fatigue and reduced perceptual–motor performance [34]. This results directly from metabolic alterations, including increased fat oxidation during exercise, and an increased non-esterified fatty acids level, resulting in an increased brain uptake of free tryptophan, which is a precursor of serotonin, responsible for feelings of tiredness and loss of motivation [35]. Similarly, the decreased carbohydrate intake for athletes is indicated as a risk for impaired performance for athletes, which results from the fact that decreased carbohydrates’ exploitation is accompanied by increased fat oxidation with its all consequences [36]. 

The nutritional recommendations for individuals with excessive body mass, associated with planned body mass reduction, are based on a reduced energy value of the diet, as recommended by the WHO [2] to be achieved by a reduction in fat and sugar intake, accompanied by physical activity. This is in agreement with results of the studies assessing the general effectiveness of various body mass reduction strategies, as a low-fat diet is characterized by the highest body mass reduction in a 6-month period, accompanied by reasonable adherence, which is associated with the possibility of reducing the low-density lipoprotein (LDL) cholesterol levels [37]. Moreover, the recent meta-analysis by Jabbour et al. [38] indicated that dietary interventions conducted for over 12 months are more effective than the usual diets in inducing weight and waist circumference loss. At the same time, the novel meta-analysis by Robinson et al. [39] indicated that decreased energy density may be an effective strategy to reduce the energy supply and the risk of excessive body mass.

In agreement with such general requirements for a low-calorie diet, in the conducted study low-calorie diets were also characterized by a lower energy value than the regular diets. Moreover, such reduced energy value was obtained by reduced fat and carbohydrates, as well as mono- and oligosaccharides’ intake, being in agreement with the recommendations by WHO [2]. However, it should be mentioned that within those recommendations, it is specified that a low-calorie diet should include increased intake of fruit, vegetables, legumes, whole grains, and nuts [2], which are associated with an increased fiber intake, which is highly recommended and associated with the effectiveness of weight loss [40]. In contrast, in the conducted study, the low-calorie diets were characterized by a lower fiber intake than the regular diets, which may result in their lower effectiveness. 

Similarly, it should be emphasized that the lower iron intake in the low-calorie diets than in the regular diets, as observed in this study, may also reduce the beneficial effects of body mass reduction. This results from the fact that food restrictions may cause inadequate iron intake, and symptoms of iron deficiency, including anemia [41], which is a common problem in young women [42]. At the same time, excessive body mass is by some authors indicated to be associated with deficiency or decreased intake of iron [43], as well as magnesium [44] and vitamin B6 [45], while in the conducted study, in the low-calorie diets, the intake of all these nutrients was reduced. Taking this into account, it should be indicated that some of the nutritional value alterations for the assessed low-calorie diets were not justified.

It is worth noting that the diets described as low-calorie were presented much more frequently in the women’s magazine than in the sports magazine. This fact shows a calorie-centered approach to nutrition typical for such magazines, commonly indicated as a problem [19]. Taking into consideration the potential lower dietary awareness among their readers, this may increase the problem of following an improperly balanced diet, including the body mass reduction in readers who do not require such reduction [20] and a diet with a decreased level of important nutrients, as was revealed in the conducted study.

The nutritional recommendations associated with the COVID-19 pandemic were developed by various authorities—described within the systematic review by de Faria Coelho-Ravagnani et al. [46], published in April 2021; eight dietary recommendations developed by nutrition societies and associations and six dietary recommendations developed by national governments were found. They were elaborated separately for individuals in various conditions—critically ill patients [47], mildly ill patients during home isolation [48], or healthy individuals to prevent COVID-19 by supporting the immune system [49]. 

The systematic review by de Faria Coelho-Ravagnani et al. [46] indicated that the guidelines emphasized the importance of minerals and vitamins such as zinc and vitamins C, A, and D to maintain a well-functioning immune system, being potentially beneficial for both infected patients and individuals at risk of COVID-19 infection, including those with nutritional deficiencies. Especially for vitamin D the results are promising, as the systematic review by Yisak et al. [50] indicated that vitamin D status may be a determinant for the risk of COVID-19 infection, as well as its course and mortality rate. This corresponds with the results of the conducted study, as the diets published during the COVID-19 pandemic were characterized by increased intake of vitamin D compared with the diets before the pandemic (4.5 µg, compared with 2.4 µg). Despite the fact that the dietary vitamin D intake was still lower than recommended [51], it was much higher than the typical vitamin D intake in Polish women [52], so it must be interpreted as an important change in the commonly promoted diets.

Similar observations were formulated for calcium, being a biomarker of clinical severity at the beginning of COVID-19 symptom onset [53], and vitamin B6, being indicated as an important factor in suppressing COVID-19 severity and ameliorating its complications [54]. For both of these, as potentially beneficial during the COVID-19 pandemic, the intake was increased in diets published in this period. Last but not least, the protein level must be indicated, as this nutrient may be crucial for critically ill patients [47]. This results from the fact that the protein intake in the early acute phase of COVID-19 infection was associated with better survival and lower risk of in-hospital mortality [55].

Taking into account the problems described above, associated with the diets presented in the Polish women’s and sports magazines, we recommend presenting the energy value and nutritional value of such diets in magazines in order to compare them with the recommended intake, as well as to clearly define the target group for the diet.

Despite the fact that the conducted analysis provided some observations associated with the nutritional value of the diets presented in the Polish women’s and sports magazines, there were some limitations of the study. The conducted study focused on the macronutrients, minerals, and vitamins, while other potentially bioactive substances were not studied. Moreover, while assessing the changes in the typical nutritional value of the diets presented in these Polish magazines during the COVID-19 pandemic, they were interpreted taking into account the current state of knowledge about the influence of diet on prevention and treatment of COVID-19; this issue is still being studied, and little is known. Last but not least, it must be emphasized that the presented analysis was conducted only for Polish magazines, presenting Polish diets, including specific products and dishes, so a similar analysis should be conducted also in other countries in order to allow for international analysis and comparisons.

## 5. Conclusions

The majority of the assessed elements of the nutritional value of the diets did not differ in the subgroups stratified by type of magazine, year of publication, and type of diet. The nutritional value of the diets presented in the Polish women’s and sports magazines was not justified by the type of magazine or type of diet, associated with the target group, which may cause the following of improperly balanced diets. However, the changes in the typical nutritional value of the diets presented in the Polish women’s and sports magazines during the COVID-19 pandemic were justified by some specific needs for the prevention and treatment of COVID-19. 

## Figures and Tables

**Table 1:** The energy value and macronutrient intake within the assessed single-day menus, stratified by type of magazine, for the diets published in the women’s magazine and the diets published in the sports magazine.

	Women’s Magazine (*n* = 41)		Sports Magazine (*n* = 78)		*p*-Value
	Mean ± SD	Median (Minimum–Maximum)	Mean ± SD	Median (Minimum–Maximum)	
Energy (kcal)	1576.44 ± 343.67	1573.18 (818.00–2546.00)	1754.66 ± 467.12	1671.50 * (922.00–4378.00)	0.0517
Protein (g)	82.53 ± 27.64	85.52 (25.59–142.32)	91.11 ± 30.97	88.27 * (29.51–224.57)	0.2358
Fat (g)	54.57 ± 22.18	49.85 * (17.51–124.98)	76.74 ± 30.49	69.44 * (31.19–186.83)	<0.0001
Carbohydrates (g)	215.88 ± 73.87	216.24 (31.21–344.15)	192.97 ± 62.36	193.30 * (53.56–467.78)	0.0226
Cholesterol (mg)	236.12 ± 181.53	202.50 * (0.00–759.20)	315.27 ± 293.81	213.95 * (0.00–1507.00)	0.3334
Fiber (g)	36.00 ± 15.92	35.01 (9.28–85.63)	32.95 ± 11.56	32.26 (6.80–62.00)	0.0163
Protein (% of energy)	20.83 ± 5.30	21.86 (10.09–31.06)	21.02 ± 5.82	20.96 (7.97–38.83)	0.5258
Fat (% of energy)	31.14 ± 11.32	27.71 * (13.42–76.43)	38.83 ± 9.32	38.52 * (24.97–63.42)	<0.0001
Carbohydrates (% of energy)	49.46 ± 13.16	50.46 * (5.96–76.74)	41.60 ± 11.61	42.14 (14.17–65.88)	0.0002
Mono- and oligosaccharides (% of energy)	13.79 ± 10.51	10.92 * (1.05–61.03)	11.62 ± 6.18	10.39 * (2.53–28.32)	0.4502

* Nonparametric distribution (the Shapiro-Wilk test; *p* ≤ 0.05).

**Table 2:** The mineral intake within the assessed single-day menus, stratified by type of magazine, for the diets published in the women’s magazine and the diets published in the sports magazine.

	Women’s Magazine (*n* = 41)		Sports Magazine (*n* = 78)		*p*-Value
	Mean ± SD	Median (Minimum–Maximum)	Mean ± SD	Median (Minimum–Maximum)	
Sodium (mg)	1525.46 ± 820.07	1409.69 * (293.30–4108.78)	1724.90 ± 1057.86	1496.55 * (71.77–5313.22)	0.4879
Potassium (mg)	4204.57 ± 1340.24	4134.75 * (1608.7–8967.65)	4093.83 ± 1170.87	4106.43 * (1614.1–7750.54)	0.6339
Calcium (mg)	712.66 ± 248.23	688.25 (317.95–1224.55)	850.62 ± 339.18	770.91 * (223.40–2173.70)	0.0330
Phosphorus (mg)	1515.63 ± 413.78	1486.95 (552.72–2367.13)	1518.37 ± 479.10	1452.13 * (501.04–3248.07)	0.6300
Magnesium (mg)	422.30 ± 131.57	410.45 (161.78–700.35)	450.65 ± 140.98	439.01 (160.15–850.75)	0.6408
Iron (mg)	14.33 ± 4.76	14.38 (5.69–26.22)	16.15 ± 5.84	15.31 * (5.95–39.68)	0.1868
Zinc (mg)	10.51 ± 2.97	10.95 (3.77–18.28)	10.70 ± 3.27	10.62 (3.70–21.95)	0.5103
Copper (mg)	1.80 ± 0.67	1.76 * (0.57–3.96)	1.91 ± 0.67	1.89 * (0.75–4.54)	0.2658
Iodine (μg)	82.76 ± 68.03	55.13 * (12.80–260.29)	57.34 ± 40.64	43.49 * (6.95–216.13)	0.0694

* Nonparametric distribution (the Shapiro-Wilk test; *p* ≤ 0.05).

**Table 3:** The vitamin intake within the assessed single-day menus, stratified by type of magazine, for the diets published in the women’s magazine and the diets published in the sports magazine.

	Women’s Magazine (*n* = 41)		Sports Magazine (*n* = 78)		*p*-Value
	Mean ± SD	Median (Minimum–Maximum)	Mean ± SD	Median (Minimum–Maximum)	
Vitamin A (μg RE)	2576.15 ± 3025.05	1856.90 * (132.6–20014.80)	1990.60 ± 1340.14	1703.98 * (144.60–7424.52)	0.2403
Vitamin D (μg)	5.17 ± 5.64	3.09 * (0.00–21.45)	6.50 ± 8.87	2.48 * (0.00–41.40)	0.7573
Vitamin E (mg a-TE)	16.89 ± 7.12	15.01 * (1.72–32.99)	16.98 ± 7.41	16.12 * (2.05–41.43)	0.9445
Vitamin B1 (mg)	1.40 ± 0.50	1.33 (0.45–3.12)	1.45 ± 0.67	1.35 * (0.49–4.50)	0.8827
Vitamin B2 (mg)	1.76 ± 0.49	1.78 (0.48–3.00)	1.82 ± 0.80	1.73 * (0.49–4.25)	0.6741
Niacin (mg)	21.22 ± 9.53	18.15 * (7.21–49.24)	23.98 ± 13.12	20.34 * (5.80–67.10)	0.3953
Vitamin B6 (mg)	2.81 ± 0.79	2.86 (1.12–4.94)	2.64 ± 1.03	2.62 * (0.55–5.82)	0.2380
Folate (μg)	538.50 ± 199.07	520.60 * (155.27–1258.35)	593.54 ± 266.63	549.34 * (88.21–1303.00)	0.3089
Vitamin B12 (μg)	4.54 ± 3.85	3.51 * (0.00–15.91)	4.38 ± 3.49	3.60 * (0.00–17.20)	0.9401
Vitamin C (mg)	315.26 ± 170.46	312.00 (35.07–776.56)	270.45 ± 157.70	262.32 * (9.94–724.7)	0.1489

* Nonparametric distribution (the Shapiro-Wilk test; *p* ≤ 0.05); RE—retinol equivalents; a-TE—α-tocopherol equivalents.

**Table 4:** The energy value and macronutrient intake within the assessed single-day menus, stratified by year of publication, for the diets published before the COVID-19 pandemic and the diets published during the COVID-19 pandemic.

	Diets Published before COVID-19 (*n* = 78)		Diets Published during COVID-19 (*n* = 41)		*p*-Value
	Mean ± SD	Median (Minimum–Maximum)	Mean ± SD	Median (Minimum–Maximum)	
Energy (kcal)	1650.29 ± 350.97	1599.66 * (885.00–2744.00)	1775.00 ± 558.50	1715.00 * (818.00–4378.00)	0.1238
Protein (g)	82.69 ± 27.84	82.87 (25.59–144.08)	98.55 ± 31.61	95.12 * (45.27–224.57)	0.0166
Fat (g)	66.66 ± 28.38	62.43 * (17.51–152.37)	73.77 ± 32.05	65.24 * (33.35–186.83)	0.2358
Carbohydrates (g)	205.69 ± 65.17	201.86 (31.21–344.15)	191.67 ± 70.63	197.32 * (64.18–467.78)	0.2042
Cholesterol (mg)	249.59 ± 209.12	196.95 * (0.00–894.40)	361.08 ± 332.87	245.90 * (0.00–1507.00)	0.0866
Fiber (g)	35.17 ± 13.72	33.65 * (6.80–85.63)	32.07 ± 12.21	31.75 (9.79–62.00)	0.2780
Protein (% of energy)	20.12 ± 5.92	20.94 (7.97–32.43)	22.54 ± 4.68	22.39 * (14.33–38.83)	0.0589
Fat (% of energy)	35.79 ± 11.66	34.05 * (13.42–76.43)	36.94 ± 8.51	36.88 * (24.97–63.42)	0.3830
Carbohydrates (% of energy)	44.79 ± 13.91	46.01 (5.96–76.74)	43.38 ± 10.01	43.86 (14.26–59.42)	0.0243
Mono- and oligosaccharides (% of energy)	11.70 ± 8.41	9.01 * (1.05–61.03)	13.64 ± 6.96	12.38 (2.53–28.32)	0.0660

* Nonparametric distribution (the Shapiro-Wilk test; *p* ≤ 0.05).

**Table 5:** The mineral intake within the assessed single-day menus, stratified by year of publication, for the diets published before the COVID-19 pandemic and the diets published during the COVID-19 pandemic.

	Diets Published before COVID-19 (*n* = 78)		Diets Published during COVID-19 (*n* = 41)		*p*-Value
	Mean ± SD	Median (Minimum–Maximum)	Mean ± SD	Median (Minimum–Maximum)	
Sodium (mg)	1524.09 ± 911.58	1368.58 * (71.77–4175.53)	1907.49 ± 1074.73	1631.35 * (291.16–5313.22)	0.0465
Potassium (mg)	4115.83 ± 1120.27	4131.19 * (1608.70–8967.65)	4162.73 ± 1423.43	4089.25 (1614.1–7750.54)	0.9489
Calcium (mg)	753.80 ± 239.40	753.92 (223.40–1346.00)	896.86 ± 414.47	764.05 (271.40–2173.7)	<0.0001
Phosphorus (mg)	1478.74 ± 420.13	1458.88 (501.04–2438.20)	1591.02 ± 514.63	1501.17 * (741.25–3248.07)	0.5794
Magnesium (mg)	433.27 ± 133.69	431.25 (161.78–820.38)	455.37 ± 146.22	437.16 (160.15–850.75)	0.4953
Iron (mg)	15.40 ± 4.46	14.65 (5.69–26.22)	15.86 ± 7.20	14.96 * (5.95–39.68)	0.8915
Zinc (mg)	10.61 ± 2.98	10.87 (3.70–18.28)	10.69 ± 3.51	10.58 (4.84–21.95)	0.2116
Copper (mg)	1.90 ± 0.67	1.82 * (0.84–4.54)	1.81 ± 0.69	1.88 (0.57–3.81)	0.8651
Iodine (μg)	71.58 ± 61.61	49.12 * (6.95–260.29)	55.67 ± 27.68	53.94 * (15.92–124.5)	0.8000

* Nonparametric distribution (the Shapiro-Wilk test; *p* ≤ 0.05).

**Table 6:** The vitamin intake within the assessed single-day menus, stratified by year of publication, for the diets published before the COVID-19 pandemic and the diets published during the COVID-19 pandemic.

	Diets Published before COVID-19 (*n* = 78)		Diets Published during COVID-19 (*n* = 41)		*p*-Value
	Mean ± SD	Median (Minimum–Maximum)	Mean ± SD	Median (Minimum–Maximum)	
Vitamin A (μg RE)	2228.85 ± 2348.74	1806.58 * (132.60–20014.80)	2119.90 ± 1486.23	1952.60 * (144.60–7424.52)	0.9047
Vitamin D (μg)	4.67 ± 6.10	2.36 * (0.00–32.10)	8.64 ± 10.11	4.50 * (0.00–41.40)	0.0197
Vitamin E (mg a-TE)	17.17 ± 6.96	15.50 (1.72–36.16)	16.52 ± 7.94	15.82 (2.05–41.43)	0.3236
Vitamin B1 (mg)	1.48 ± 0.65	1.36 * (0.45–4.50)	1.36 ± 0.53	1.29 (0.49–2.86)	0.3591
Vitamin B2 (mg)	1.77 ± 0.64	1.73 (0.48–3.63)	1.87 ± 0.81	1.73 * (0.97–4.25)	0.9578
Niacin (mg)	21.52 ± 10.37	18.62 * (5.80–50.37)	25.91 ± 14.40	20.16 * (5.92–67.10)	0.1457
Vitamin B6 (mg)	2.61 ± 0.84	2.62 (0.55–4.94)	2.87 ± 1.14	2.93 (0.71–5.82)	0.0207
Folate (μg)	580.53 ± 224.40	548.00 * (155.27–1258.35)	563.25 ± 285.27	524.35 * (88.21–1303.00)	0.4985
Vitamin B12 (μg)	4.00 ± 3.54	3.37 * (0.00–15.91)	5.28 ± 3.61	4.66 * (0.00–17.20)	0.0277
Vitamin C (mg)	298.48 ± 161.30	273.78 * (35.07–776.56)	261.91 ± 165.21	259.98 * (9.94–724.7)	0.1887

* Nonparametric distribution (the Shapiro-Wilk test; *p* ≤ 0.05); RE—retinol equivalents; a-TE—α-tocopherol equivalents.

**Table 7:** The energy value and macronutrient intake within the assessed single-day menus, stratified by type of diet, for the regular diets (not presented as low-calorie) and the diets presented as low-calorie.

	Regular Diets (*n* = 61)		Low-Calorie Diets (*n* = 58)		*p*-Value
	Mean ± SD	Median (Minimum–Maximum)	Mean ± SD	Median (Minimum–Maximum)	
Energy (kcal)	1815.90 ± 510.77	1715.00 * (922.00–4378.00)	1564.27 ± 291.51	1574.55 * (818.00–2546.00)	0.0020
Protein (g)	89.39 ± 33.41	89.36 * (29.51–224.57)	86.85 ± 26.24	85.83 (25.59–144.08)	0.8260
Fat (g)	77.58 ± 34.75	65.83 * (31.19–186.83)	60.20 ± 20.10	58.16 (17.51–106.28)	0.0162
Carbohydrates (g)	207.87 ± 75.20	201.22 (31.21–467.78)	193.49 ± 57.19	193.57 (72.24–344.07)	0.0390
Cholesterol (mg)	328.12 ± 323.56	216.00 * (0.00–1507.00)	245.81 ± 170.15	201.00 * (1.50–759.20)	0.5618
Fiber (g)	34.44 ± 15.02	33.19 (6.80–85.63)	33.75 ± 11.22	32.39 (9.79–60.05)	<0.0001
Protein (% of energy)	19.87 ± 5.86	20.52 (7.97–38.83)	22.09 ± 5.18	22.17 (10.09–32.43)	0.3529
Fat (% of energy)	37.87 ± 11.71	35.63 * (21.48–76.43)	34.41 ± 9.19	34.94 (13.42–53.77)	0.3305
Carbohydrates (% of energy)	37.53 ± 19.88	44.02 * (0.06–65.88)	43.82 ± 12.44	43.05 (15.64–76.74)	0.4189
Mono- and oligosaccharides (% of energy)	13.66 ± 6.97	12.92 (1.14–28.32)	11.01 ± 8.75	8.92 * (1.05–61.03)	0.0084

* Nonparametric distribution (the Shapiro-Wilk test; *p* ≤ 0.05).

**Table 8:** The mineral intake within the assessed single-day menus, stratified by type of diet, for the regular diets (not presented as low-calorie) and the diets presented as low-calorie.

	Regular Diets (*n* = 61)		Low-Calorie Diets (*n* = 58)		*p*-Value
	Mean ± SD	Median (Minimum–Maximum)	Mean ± SD	Median (Minimum–Maximum)	
Sodium (mg)	1666.83 ± 991.93	1511.87 * (291.16–5313.22)	1644.99 ± 983.08	1383.40 * (71.77–4175.53)	0.8260
Potassium (mg)	4236.73 ± 1496.31	4134.75 * (1608.70–8967.65)	4021.83 ± 858.72	4108.75 (1650.25–5861.82)	0.8260
Calcium (mg)	818.37 ± 379.30	753.13 * (223.40–2173.7)	787.02 ± 235.94	767.39 (338.70–1346.00)	0.9304
Phosphorus (mg)	1505.65 ± 508.24	1410.63 * (501.04–3248.07)	1529.81 ± 397.53	1519.00 (552.72–2438.20)	0.3549
Magnesium (mg)	470.92 ± 151.97	486.85 (160.15–850.75)	409.30 ± 114.40	403.61 (161.78–700.35)	0.0323
Iron (mg)	16.58 ± 6.00	15.90 * (5.95–39.68)	14.48 ± 4.81	14.13 * (5.69–33.39)	0.0307
Zinc (mg)	10.55 ± 3.32	10.65 (3.70–21.95)	10.72 ± 3.00	10.84 (3.77–18.28)	0.4338
Copper (mg)	1.97 ± 0.80	1.96 * (0.75–4.54)	1.77 ± 0.49	1.80 (0.57–3.25)	0.2315
Iodine (μg)	61.42 ± 47.94	47.69 * (6.95–222.72)	71.03 ± 57.59	51.57 * (12.80–260.29)	0.3439

* Nonparametric distribution (the Shapiro-Wilk test; *p* ≤ 0.05).

**Table 9:** The vitamin intake within the assessed single-day menus, stratified by type of diet, for the regular diets (not presented as low-calorie) and the diets presented as low-calorie.

	Regular Diets (*n* = 61)		Low-Calorie Diets (*n* = 58)		*p*-Value
	Mean ± SD	Median (Minimum–Maximum)	Mean ± SD	Median (Minimum–Maximum)	
Vitamin A (μg RE)	2331.21 ± 2752.15	1692.24 * (144.6–20,014.8)	2044.18 ± 995.97	1865.28 (132.60–4891.30)	0.3774
Vitamin D (μg)	6.85 ± 9.09	3.12 * (0.00–41.40)	5.19 ± 6.41	2.35 * (0.00–24.60)	0.3227
Vitamin E (mg a-TE)	18.17 ± 8.27	17.91 (2.05–41.43)	15.66 ± 5.88	14.77 * (1.72–32.99)	0.1189
Vitamin B1 (mg)	1.38 ± 0.51	1.34 * (0.49–3.12)	1.50 ± 0.71	1.34 * (0.45–4.50)	0.3978
Vitamin B2 (mg)	1.77 ± 0.78	1.63 * (0.49–4.25)	1.83 ± 0.61	1.78 (0.48–3.63)	0.2253
Niacin (mg)	23.19 ± 12.88	19.88 * (5.80–67.10)	22.86 ± 11.19	18.57 * (7.21–50.37)	0.9641
Vitamin B6 (mg)	2.73 ± 1.09	2.63 (0.55–5.82)	2.67 ± 0.80	2.67 (0.80–4.81)	0.0204
Folate (μg)	577.53 ± 271.51	554.70 * (88.21–1303)	571.47 ± 218.30	524.79 * (155.27–1173.70)	0.8968
Vitamin B12 (μg)	4.93 ± 4.39	3.92 * (0.00–17.20)	3.92 ± 2.45	3.53 * (0.10–11.70)	0.6018
Vitamin C (mg)	261.35 ± 168.45	224.02 * (9.94–724.70)	311.68 ± 154.09	301.61 * (77.20–776.56)	0.0524

* Nonparametric distribution (the Shapiro-Wilk test; *p* ≤ 0.05); RE—retinol equivalents; a-TE—α-tocopherol equivalents.
